# Biocarbon Meets Carbon—Humic Acid/Graphite Electrodes Formed by Mechanochemistry

**DOI:** 10.3390/ma12244032

**Published:** 2019-12-04

**Authors:** Lianlian Liu, Niclas Solin, Olle Inganäs

**Affiliations:** Biomolecular and organic electronics, Department of Physics, Chemistry and Biology, Linköping University, 58183 Linköping, Sweden; lianlian.liu@liu.se (L.L.); niclas.solin@liu.se (N.S.)

**Keywords:** mechanochemistry, humic acid, graphite, biocarbon, energy storage

## Abstract

Humic acid (HA) is a biopolymer formed from degraded plants, making it a ubiquitous, renewable, sustainable, and low cost source of biocarbon materials. HA contains abundant functional groups, such as carboxyl-, phenolic/alcoholic hydroxyl-, ketone-, and quinone/hydroquinone (Q/QH_2_)-groups. The presence of Q/QH_2_ groups makes HA redox active and, accordingly, HA is a candidate material for energy storage. However, as HA is an electronic insulator, it is essential to combine it with conductive materials in order to enable fabrication of HA electrodes. One of the lowest cost types of conductive materials that can be considered is carbon-based conductors such as graphite. Herein, we develop a facile method allowing the biocarbon to meet carbon; HA (in the form of a sodium salt) is mixed with graphite by a solvent-free mechanochemical method involving ball milling. Few-layer graphene sheets are formed and the HA/graphite mixtures can be used to fabricate HA/graphite hybrid material electrodes. These electrodes exhibit a conductivity of up to 160 S·m^−1^ and a discharge capacity as large as 20 mAhg^−1^. Our study demonstrates a novel methodology enabling scalable fabrication of low cost and sustainable organic electrodes for application as supercapacitors.

## 1. Introduction

In order to reduce the consumption of fossil-based energy sources, and thereby decrease the release of carbon dioxide, there is a great need for increased use of renewable energy sources. Indeed, energy captured from both the sun and wind are currently widely utilized for electricity generation; however, the intermittent nature of solar and wind power means that energy storage systems are required in order to supply a stable energy over time. This aspect is especially important for the regions that lack a distributed power grid. In such areas—typically around the equator on the African continent—local electricity sources powered by the sun in combination with local electricity storage provide an attractive option [1]. It should be noted that large-scale introduction of renewable energy sources thus needs to be coupled with large-scale capacity for energy storage. It is thus essential to not only develop sustainable sources of electrical energy, but also devices for charge storage based on scalable and low cost materials whose extraction has a low impact on the environment.

An outstanding source of such materials would be plants. Plants grow on the surface of the earth and have been utilized for thousands of years in all known human civilizations. Moreover, many plant derived materials contain a sizable fraction of redox active quinone (Q) groups. A famous example is lignin, one of the most abundant biopolymers. Lignin has recently been employed as a key component of charge storage devices, where charge is stored in the redox active groups: quinone/hydroquinone (Q/QH_2_) [2,3,4]. Humic acid (HA) is structurally closely related to lignin, and also contains a large fraction of Q/QH_2_ groups. HA and related biomass materials are some of the most widely distributed natural polymers on earth. The source of HA is mainly the degradation of organic matter and HA is present in a variety of forms and environments, such as soil, natural waters, sea sediment, peat, lignite, and coal [5,6,7,8]. HA consists of cross-linked substituted aromatic rings and a wide variety of oxygen-containing functional groups, such as carboxylic acids, phenolic and alcoholic hydroxyls, ketones, and Q/QH_2_ groups [6,8,9], which can perform redox reactions and, accordingly, make it a promising material for energy storage.

Currently the main applications of HA involve the synthesis of active carbon [5] and porous carbon [9], removal of heavy metals [10] and waste gases [11], as well as for antioxidants [8]. The redox activity of Q/QH_2_ groups in HA has been previously studied [12,13,14]; however, HA is challenging to employ for charge storage because it is an electronic insulator. Zhu utilized HA as anodes for lithium ion btteries [15], Wasinski applied HA as electrolyte additive to improve the discharge capacity of double layer capacitors [16]. However, in order to apply HA in organic electrodes and obtain efficient charge storage, it is essential to contact the redox active groups in HA with conductive materials. As pointed out earlier, due to the importance of large-scale production of charge storage devices it is essential that low-cost conductive materials, for example carbon based materials such as graphite, are employed. Graphite electrodes are widely utilized in batteries [17,18], fuel cell [19] and electrolysis [20]. Moreover, graphite can be converted into a carbon paste that can in turn be processed into electrodes [21]. 

Pristine graphite, built up from stacks of individual graphene sheets, displays a conductivity ranging between 100–500 S·m^−1^ ꓕ (perpendicular to the planes), 2–2.5 × 10^6^ S·m^−1^ || (parallel to graphene layer) [22]. It can be exfoliated into stacks of graphene sheets with different numbers of layers including single layer graphene. A wide variety of methods have been developed to exfoliate graphite into graphene sheets including aqueous assisted sonication [23,24] and mechanochemistry (grinding) [25]. Keeping the importance of scalability in mind, mechanical milling is an especially attractive method to exfoliate graphite with the help of surfactants [26,27]. Herein, we report a facile process to make the biocarbon HA meet the carbon material graphite: HA and graphite are co-milled in the solid state by means of a ball-mill; the resulting solid mixture is then treated with water, leading to formation of a paste that is coated onto substrates and thereafter can be applied in energy storage devices. It should be noted that dependent on the pH, HA will be present in a protonated or deprotonated form. At higher pH a HA salt is formed that will have a high solubility in water. Herein, HA is employed in the form of a sodium salt. 

To the best of our knowledge, HA has not been combined with graphite for application in charge storage devices. The employment of mechanochemical process in the solid state without any other additive makes it promising for scalable fabrication of the HA/graphite electrodes. We find that the fabricated HA/graphite hybrid electrodes show conductivity of 160 S·m^−1^ and discharge capacity of 20 mAhg^−1^, with a specific capacitance of 60 F·g^−1^, which is much higher than the reported graphite electrode (5 F·g^−1^) [28], carbon black (12.6 F·g^−1^) [29] and the single-walled carbon nanotube (18 F·g^−1^) [30]. The discharge capacity of the HA/graphite hybrid electrodes is lower than that for lignin electrodes (see the comparison in Table 1). However, HA is an abundant biopolymer and the results reported herein increase the variety of biomass materials that can be considered for energy storage. Thus the combination of the biocarbon (HA) and carbon (graphite) by mechanochemistry enables the fabrication of sustainable, scalable, and low cost energy storage devices.

## 2. Materials and Methods 

### 2.1. Materials

#### 2.1.1. Chemical and Reagents 

Humic acid sodium salt (Sigma-Aldrich, Steinheim, Germany), graphite (flakes, mp 3652–3697 °C, density 1.9, Sigma-Aldrich, Saint Louis, MO, USA), perchloric acid (HClO_4_) (Sigma-Aldrich, Steinheim, Germany) and KBr (Scharlau, Barcelona, Spain) were utilized as received. The aqueous solutions were prepared with ultrapure deionized water (Millipore).

#### 2.1.2. Mechanical Grinding

The HA/graphite mixture is prepared by a mixer mill MM400 (Retsch, Haan, Germany). A stainless steel cup of 1.5 mL, stainless steel balls with a diameter of 3 mm, a frequency of 30 Hz, and a milling time of 99 min are employed during the ball milling process. Additionally, 20 milling balls (with a total weight of 2.2 g) and approximate 120 mg of the samples are filled into the milling cup to have enough free space for the efficient shear force for the exfoliation of graphite. Herein, we apply different weight ratio of HA and graphite: x/1 (x = 2, 4, 7, 10, w/w), during the milling process. The collected mixtures of HA/graphite with different stoichiometry after milling are labeled as HA/graphite (x/1, w/w) mixture.

#### 2.1.3. Electrode Preparation 

100 mg of the above milling mixture was dispersed into 1 mL of distilled water by means of treatment of a vortex shaker (REAX 2000 KEBO-Lab, Spånga, Sweden) for 4 min. The dispersion was then centrifuged (universal 320 R, Hettich, Tuttlingen, Germany) at 4000 rpm for 2 min, in order to remove large graphite particles. Secondly, the supernatant was collected and further centrifuged for 70 min at 6000 rpm. Then the pellets were collected and painted on the gold electrodes via an automatic blade-coater (with a ZFR 2040 film applicator, 30 µm gap, and 15 mm/s blade speed, Erichsen 510, Hemer, Germany). Before running the other measurements, the hybrid electrodes were dried at 120 °C for 10 min to remove water. Throughout the text below, the materials obtained from the pellets are designated as HA/graphite (x/1, w/w) hybrid material pellets, and the electrodes are designated as HA/graphite (x/1, w/w) hybrid material electrodes (here, “x” refers to the weight ratio of HA and graphite in the milling step).

### 2.2. Methods

#### 2.2.1. Electrochemical Measurements 

A standard three-electrode configuration, with a platinum wire as counter electrode (CE), a Ag/AgCl (KCl salt) as reference electrode (RE) and materials coated onto gold (evaporated onto a silicon wafer 4″ (1.0.0)) as working electrode (WE) (Bioanalytical Systems Inc., West Lafayette, Indiana, USA), was employed for electrochemical measurements. Cyclic voltammograms (CVs), cycling stability, and galvanostatic charge-discharge cycles were investigated via Autolab PGStat 10 (EchoChemie, the Netherlands). For CVs measurements, the potential window was cycled from −0.4 to 0.9 V (vs. Ag/AgCl) for 20 cycles at 50 mV/s. Different charge rates (0.1–16 A/g) were utilized for galvanostatic charge-discharge cycles. The cycling stability measurements were run at a potential range from −0.3 to 0.9 V (vs. Ag/AgCl) and a charge rate of 1 A/g. The discharge capacity is defined by Equation (1):

*Q* = *I* ∆*t*/*m* (1)

where *Q* is the discharge capacity, *I* is the charge-discharge current, ∆*t* is the discharging time, and *m* is the mass of the hybrid materials.

#### 2.2.2. Thermogravimetric Analysis (TGA) 

The thermal stability of the hybrid materials and the mass ratio of HA and graphite in the hybrid material pellets were performed with STA 449 F1 Jupiter thermal analysis (NETZSCH, Selb, Germany). The samples were heated from room temperature to 1000 °C with an increasing speed of 10 °C/min under Argon.

#### 2.2.3. Fourier Transform Infrared Spectroscopy (FTIR)

Fourier transform infrared spectroscopy (FTIR) spectra were recorded in transmittance mode on a VERTEX (Bruker, Billerica, MA, USA) at room temperature. The graphite flakes, HA powder, and the HA/graphite hybrid material pellets powder (after drying) were ground with KBr and pressed into KBr-pellets for the measurements. 

#### 2.2.4. Scanning Electron Microscopy (SEM).

Scanning electron microscopy (SEM) images were acquired via Leo 1550 Gemini Scanning Electron Microscope with an acceleration voltage of 5 kV (Zeiss, Oberkochen, Germany). The HA/graphite hybrid material pellets were coated onto gold substrates for the measurements. 

#### 2.2.5. Raman Spectroscopy

Raman spectroscopy was recorded via a home assembled Raman instrument equipped with a spectrograph (Kymera 328i) (ANDOR, Belfast, Northern Ireland), a microscope (Nikon eclipse Ti), and a 50x air objective with LEICA N.A. 0.75. For the preparation of the samples, the HA/graphite hybrid material pellets were coated and the HA solution was dropped onto Si wafers. An excitation laser wavelength of 532 nm and a thermoelectrically cooled Electron Multiplying charge-coupled device (EMCCD) camera (−60 °C, newton, ANDOR) were employed for the measurements. The spectra were acquired with a laser power of 31.4 mW and an integration time of 10 s on Andersolis software, in six randomly selected areas of the dry samples. The six spectra for each sample were averaged, smoothed, and baseline corrected. 

#### 2.2.6. X-ray Diffraction (XRD)

XRD was run on a X’Pert Pro diffractometer (PANalytical, Almelo, Netherlands), with Cu Kα radiation (45 KV and 40 mA). The HA/graphite hybrid material pellets were coated onto Si substrates for the measurements.

## 3. Results and Discussions

**Figure 1:**
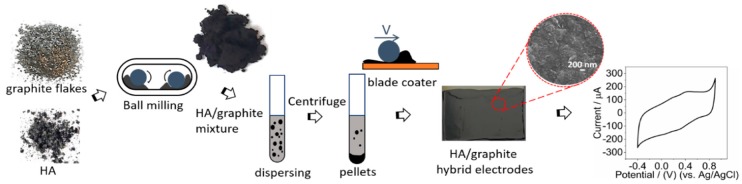
The process of the fabrication of HA/graphite hybrid material electrodes.

In order to fabricate carbon paste electrodes, cross-linkers or binders are commonly utilized [21]. Here we form a material without employing any cross-linker or binder or organic solvent to fabricate the electrodes. First of all, we disperse the mixture into water. However, it is problematic to fabricate electrodes from this dispersion by drop-casting, as it results in films with cracks upon drying. In order to avoid this problem, we separate the mixture into a pellet and a supernatant by centrifuging the as-prepared dispersion. The films formed from the supernatant still show unsatisfactory characteristics and exhibit separate aggregates instead of continuous films. However, the paste obtained from the pellet can form a continuous film without cracks, which moreover importantly does not dissolve when applying the aqueous electrolyte. Accordingly, for successful electrode preparation, we discard the material in the supernatant, and the pellet material is collected and coated onto gold electrodes via an automated blade coating technique (Figure 1). 

### 3.1. FTIR

**Figure 2:**
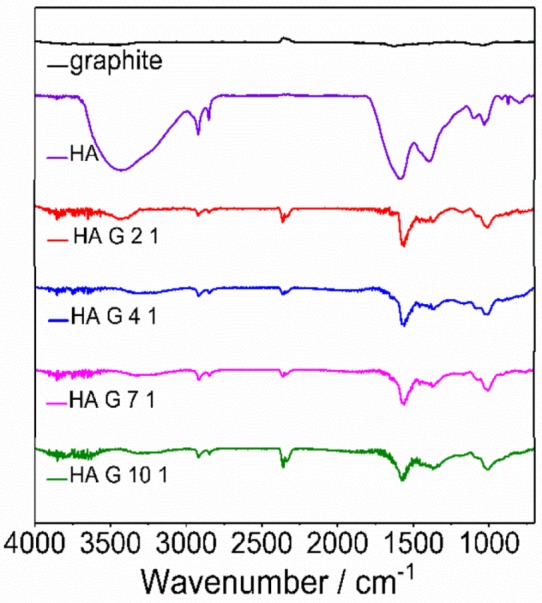
FTIR transmittance spectra of graphite, HA, HA/graphite (2/1, 4/1, 7/1, 10/1, w/w). The spectra have been baseline corrected and shifted to allow visualization.

The FTIR spectra of graphite, HA and HA/graphite hybrid material pellets with different stoichiometry are shown in Figure 2. In the spectrum of the pure HA, the peak at 3425 cm^−1^ is attributed to the stretch vibration of hydroxyl group; the peaks at 2919 and 2847 cm^−1^ are due to the vibration of -C-H_3_ in aliphatic structures; the strong absorption bands at 1583 cm^−1^ and 1392 cm^−1^ relate to the vibration of aromatic rings. Additionally, the peaks at 1100 cm^−1^ and 1032 cm^−1^ correspond to C-O stretching vibration in phenol, ether or alcohol groups. It is consistent with the reported data [6,16]. There are no peaks in the spectrum of graphite since graphite lacks functional groups. The HA/graphite hybrid material electrodes all exhibit bands at 2920 cm^−1^, 2848 cm^−1^, 1566 cm^−1^, 1080 cm^−1^, 1010 cm^−1^, and a weak absorption at 1369 cm^−1^, corresponding to the spectrum of HA. These results illustrate that there are phenolic and aromatic functional groups in HA structures, and HA exists in the HA/graphite hybrid material pellets. Moreover, the blackish color of the pellets indicates the presence of graphite (Figure 1).

### 3.2. Raman

**Figure 3:**
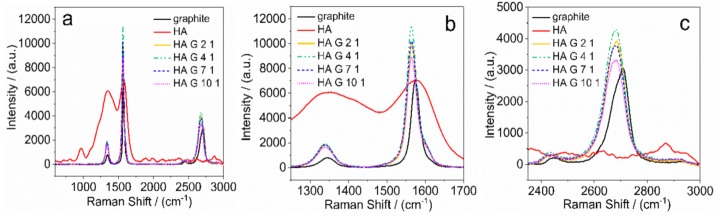
Raman spectra of HA/graphite hybrid materials with different primary stoichiometry, with 31.5 mW (532 nm) laser excitation energy (**a**–**c**).

Raman spectroscopy is employed for the geometry and electronic structures characterization of graphite, HA and the HA/graphite hybrid material electrodes. In Figure 3a, the pristine graphite presents bands at 1348 cm^−1^, 1575 cm^−1^, 2710 cm^−1^, and a tiny band at 2450 cm^−1^, due to the D band, G band, 2D band, and the two-phonon double resonance Raman scattering, respectively [27]. HA shows bands at 1350 cm^−1^ and 1576 cm^−1^, which overlap with the bands of the pristine graphite, and agree with the published data for HA [33,34]. It is, accordingly, difficult to distinguish between the bands from the pristine graphite and HA in the Raman spectra of the HA/graphite hybrid material electrodes. In addition, the bands of HA exhibit higher intensity than that of the pristine graphite and the former displays several waves on the baseline, such as the peak at 966 cm^−1^, ascribed to the fluorescence from HA. As for the HA/graphite hybrid material electrodes, they present bands at similar shift: 1340 cm^−1^, 1560 cm^−1^ 2683 cm^−1^, and a small band at 2435 cm^−1^, in agreement with the Raman shift of graphite. Comparing with the vibration of the pristine graphite, the hybrid materials, however, display a blue shift of 15 cm^−1^ and a shoulder peak at approximate 1605 cm^−1^ of the G bands (Figure 3b), a blue shift of 27 cm^−1^ of the 2D bands (Figure 3c), indicating decreasing number of layers and increasing disorder of the graphene sheets in the hybrid materials [35]. Moreover, the intensity ratio of D and G bands, *I*_D_/*I*_G_, for the HA/graphite (2/1, 4/1, 7/1, 10/1, w/w) hybrid material electrodes are 0.18, 0.17, 0.18, and 0.19, respectively, bigger than that of pristine graphite, 0.11, indicating smaller crystallite size and more disorder in the hybrid materials than the pristine graphite [36,37,38]. To conclude, the Raman results suggest that few-layer graphene sheets with smaller crystallite size, more defects and more serious disorder than the pristine graphite are found in the HA/graphite hybrid material electrodes.

### 3.3. Thermal Gravimetric Analysis (TGA)

**Figure 4:**
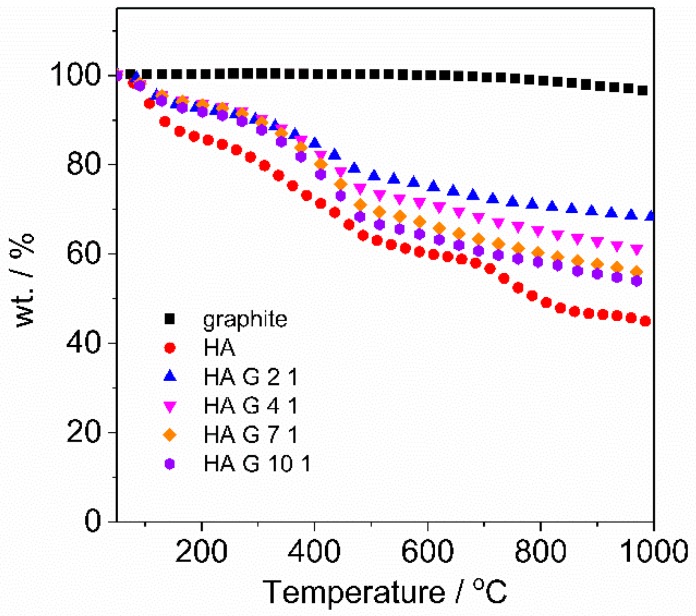
Mass change of HA, graphite, HA/graphite (2/1, 4/1, 7/1, 10/1, w/w) pellets vs. temperature in TGA curves. The measurements were obtained at a heating rate of 10 °C min^−1^ under Ar atmosphere.

TGA is applied to investigate the thermal stability and decomposition of HA during heating, and the stoichiometry of the HA/graphite hybrid material pellets after centrifugation. Graphite flakes, HA powder, and the HA/graphite hybrid material pellets powder (after drying) were heated at a heating rate of 10 °C min^−1^ under Ar atmosphere. The weight loss curve of the samples are shown in Figure 4, related to the degradation process of the materials. Graphite keeps stable during the heating process—from room temperature until 1000 °C—indicating high thermal stability. The degradation processes of HA, however, are divided into three steps: the weight loss at 50–175 °C is due to the moisture evaporation; 300 to 500 °C corresponds to the oxidation of the carbon chain and the removal of phenolics, alcohols, and aldehyde acids, along with the gaseous species, such as CO and CO_2_; 720 to 830 °C corresponds to the decomposition of aromatic rings in HA structures [39]. The weight loss curves of the HA/graphite hybrid material pellets exhibit a similar trend with that of HA as there is HA embedded in the electrodes. Furthermore, graphite, HA, the HA/graphite (2/1, 4/1, 7/1, 10/1, w/w) hybrid material pellets present residual mass fractions of 96.0%, 44.5%, 68.2%, 60.5%, 55.2%, and 53.3%, respectively, based on that, the stoichiometry of the HA/graphite hybrid material pellets are calculated (see Supplementary Materials) and exhibited in Table 2. The calculated stoichiometry of the HA/graphite (2/1, 4/1, 7/1, 10/1, w/w) hybrid material pellets are 1.17, 2.21, 3.78, and 4.85, respectively, increasing along with the mass ratio of HA/graphite in the primary mixture. Thus a larger mass fraction of HA in the primary milling process leads to a larger mass fraction of HA in the hybrid material electrodes.

### 3.4. SEM

**Figure 5:**
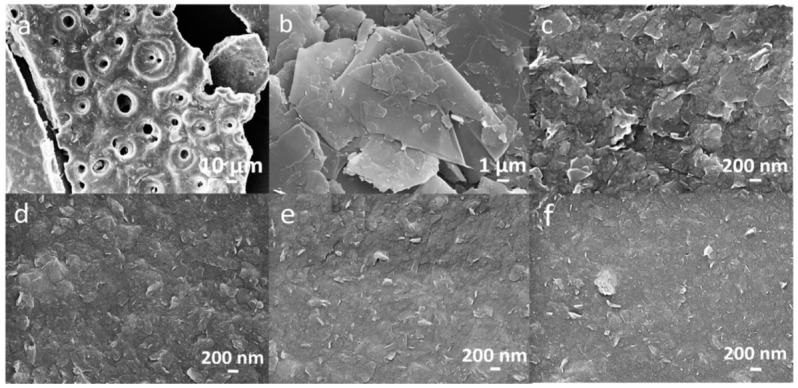
SEM images of (**a**) HA, (**b**) graphite, (**c**) HA/graphite (2/1, w/w), (**d**) HA/graphite (4/1, w/w), (**e**) HA/graphite (7/1, w/w), and (**f**) HA/graphite (10/1, w/w) hybrid material electrodes.

The morphology of HA, pristine graphite, and the HA/graphite (2/1, 4/1, 7/1, 10/1, w/w) hybrid material electrodes are investigated with SEM and the images are exhibited in Figure 5. We note that HA shows a porous structure with a particle size larger than 100 µm (Figure 5a) and the pristine graphite displays multilayer structures with a particle size of 10–20 µm (Figure 5b). For the other HA/graphite hybrid material electrodes, a much smaller particle size range, from 200 nm to 500 nm, is observed. Interestingly, HA/graphite (2/1, w/w) hybrid material electrode exhibits a larger particle size (~500 nm, Figure 5c) than the other hybrid material electrodes (~200 nm, Figure 5d–f). Moreover, when the mass ratio of HA/graphite is larger in the milling process, the grain size of the hybrid material electrode is smaller, as shown in Figure 5d–f. Accordingly, the grain size of the pristine graphite and HA are reduced during the ball milling process, leading to nanoscale geometries in the hybrid material electrodes, and different stoichiometry of HA and graphite results in different morphology of the hybrid material electrodes. 

### 3.5. XRD

**Figure 6:**
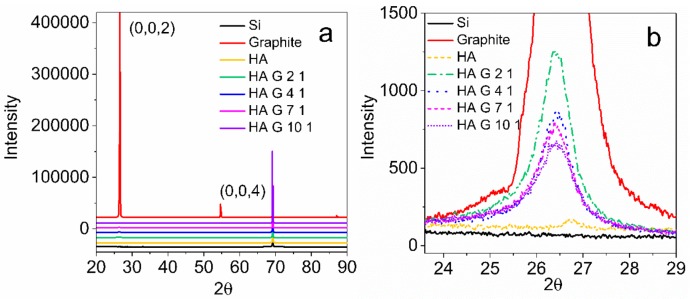
XRD patterns of HA, graphite, HA/graphite (2/1, 4/1, 7/1, and 10/1, w/w) hybrid material electrodes (**a**,**b**).

The structure of HA, graphite, and HA/graphite hybrid material electrodes are further characterized by XRD (see Figure 6). Pristine graphite presents characteristic peaks at 26.5° (0,0,2) and 54.6° (0,0,4), indicating an interlayer spacing of 0.34 nm [40]. HA exhibits very small diffuse 2ϴ peak at 26.8°, ascribed to its non-crystalline nature [41]. Moreover, the HA/graphite hybrid material electrodes display merely one peak at 26.4°, that are much smaller than the peak at 26.5 ° of graphite, illustrating graphite being exfoliated into few-layer graphene sheets with an unchanged inter-planar distance of 0.34 nm in the hybrid material electrodes [40,42]. In addition, the full width at half maximum (FWHM) of the peaks at 26.4° in graphite and the hybrid materials, which is inversely-proportional to the crystallite size of the samples, are calculated. The calculated FWHM of the HA/graphite (2/1, 4/1, 7/1, 10/1, w/w) hybrid material electrodes exhibit as 0.87°, 0.91°, 0.98°, and 1.15°, respectively, increasing slightly along with the mass ratio of HA and graphite, and they are, apparently, much larger than that of the pristine graphite (0.15°). This demonstrates that the large mass ratio of HA and graphite in the milling process results in small crystallite size of graphite in the hybrid material electrodes, and the HA/graphite hybrid material electrodes have much smaller crystallite size when comparing with the pristine graphite [43]. It is consistent with the results of SEM.

The above results unveil that HA helps to exfoliate the pristine graphite into small crystals and few-layer graphene sheets. It suggests that HA acts as a surfactant to reduce the surface tension of graphite through the shear force during the mechanochemical process. The achieved nanoscale geometry of the hybrid materials results in a short distance between the graphite and the Q/QH_2_ groups in HA, that improves the transfer kinetic of the electrons in the hybrid materials.

### 3.6. Electrochemical Measurements

Electrochemical measurements are employed to clarify the suitability of the hybrid materials for energy storage as organic electrodes. The electrochemical experiments are run with a standard three electrode system; Ag/AgCl (1 M KCl salt solution) as reference electrode (RE), platinum plate as counter electrode (CE), and the working electrodes (WE) are prepared by coating the HA/graphite hybrid materials on top of gold substrates.

**Figure 7:**
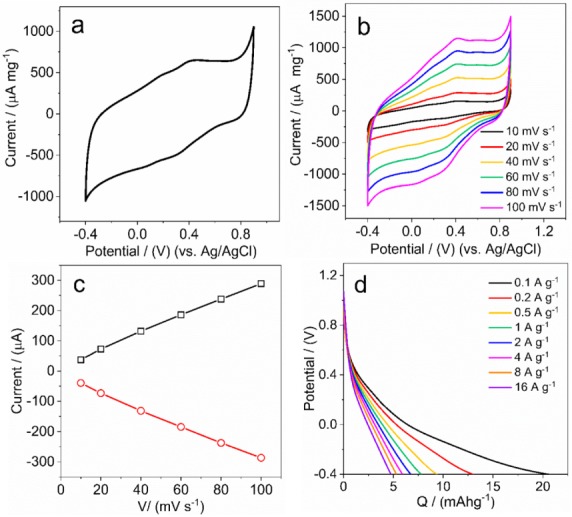
(**a**) CVs at 0.05 V/s, (**b**) CVs at different scan rate at the potential range of −0.4 V and 0.9 V, (**c**) dependence of the redox peak currents on scan rate and (**d**) galvanostatic discharge curves at the charge rate of 0.1 A·g^−1^, 0.2 A·g^−1^, 0.5 A·g^−1^, 1 A·g^−1^, 2 A·g^−1^, 4 A·g^−1^, 8 A·g^−1^, and 16 A·g^−1^ of the HA/graphite (4/1, w/w) hybrid material electrodes, in 0.1 M HClO_4_.

The CVs of HA/graphite hybrid material electrodes reveal no significantly obvious peaks in Figure 7a, but two groups of tiny and symmetric bands at 0.18 V and 0.10 V, and 0.42 V and 0.30 V, respectively, versus Ag/AgCl. We assume these bands originate from the redox reactions of the Q/QH_2_ groups in HA. There are two processes occurring during the redox reactions: the Q groups are reduced into the monomer hydroquinone (QH) form in the region of 0.39–0.24 V; the QH form is further reduced into the hydroquinone (QH_2_) form in the 0.16–0 V region. Vice versa, the QH_2_ form is oxidized into the QH form in the region of 0.1–0.24 V and further oxidized into the Q form in the 0.33–0.53 V region. We hypothesize that the graphite particles in the hybrid materials form a network that allows electrons to transport between the Q/QH_2_ groups and the current collector.

In addition, the presence of protons in the electrolyte is significant in order to form QH and QH_2_ as well as balance the charges in the HA/graphite hybrid material electrodes during the redox reactions. In order to understand the dominating electrochemical process in the electrodes, we investigate the dependence of the redox peak current on scan rate. We note that the redox peaks separation in CVs does not increase with the increasing scan rate (Figure 7b) and the peak currents are proportional to the scan rate (Figure 7c). It suggests that the redox reactions of the Q/QH_2_ groups within the electrodes are reversible [44], moreover, the electroactive species are confined and immobilized at the surface of the electrodes, where the redox processes are not limited by diffusion. We hypothesize, accordingly, that the amount of Q/QH_2_ groups in HA is small, which is consistent with the small redox peaks in the CVs, and that the protons absorbed at the surface of the electrode are sufficient for the redox reactions. 

Moreover, the discharge capacity of the HA/graphite hybrid material electrode is addressed by the galvanostatic discharge measurements. As shown in Figure 7d, the discharge curve of the HA/graphite (4/1, w/w) hybrid material electrode ends at 20 mAhg^−1^ at a charge rate of 0.1 A·g^−1^ in the potential window from −0.4 to 0.9 V, vs. Ag/AgCl. It decreases, however, to merely 5 mAhg^−1^ when applying the charge rate of 16 A·g^−1^. Furthermore, no obvious plateaus are found in the discharge curves of the HA/graphite (4/1, w/w) hybrid material electrode, however, there are different slopes in these curves, ascribed to the contributions to the capacity from different processes: the double layer capacity from graphite; the faradic capacity from the reduction of Q into QH groups (at a potential of ~0.4 V, vs. Ag/AgCl) and reduction of QH groups into QH_2_ groups (at a potential of ~0 V, vs. Ag/AgCl), respectively. It is difficult to distinguish the faradaic capacity from the double layer capacity, which is in agreement with the CVs in Figure 7a. Herein, we assume it originating from the small amount of Q/QH_2_ groups in HA, related to the results of the dependence of the redox peak current on scan rate. For the hybrid material electrodes with other stoichiometry, the discharge capacities are presented in Table 2, showing 16.7 mAhg^−1^, 20.7 mAhg^−1^, 20.1 mAhg^−1^, and 18.9 mAhg^−1^ for HA/graphite (2/1, 4/1, 7/1, 10/1, w/w) hybrid material electrodes, respectively. It indicates that the discharge capacity of the hybrid material electrodes are not affected strongly by their stoichiometry. 

**Figure 8:**
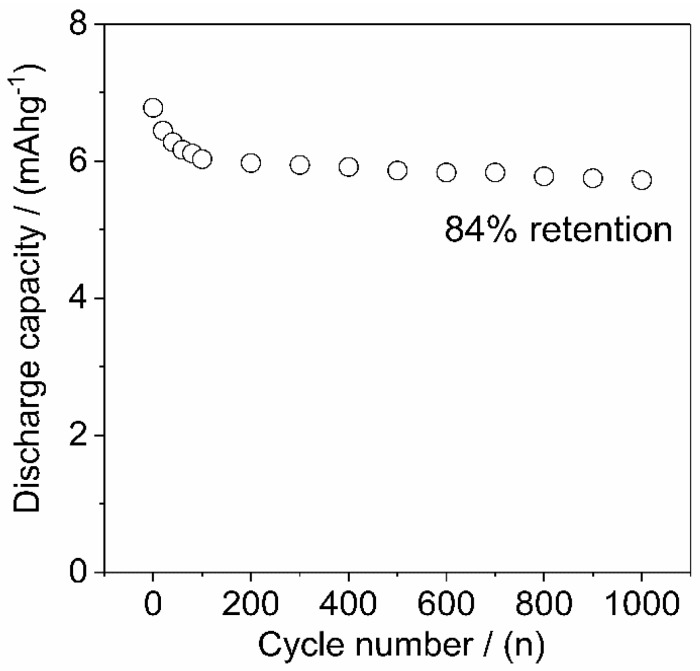
Discharge capacity after reversible charge–discharge cycles at 1 A·g^−1^ of the HA/graphite (4/1, w/w) hybrid material electrode in 0.1 M HClO_4_.

Additionally, the charge–discharge cycling stability of the HA/graphite (4/1, w/w) hybrid material electrode is investigated and the discharge capacity versus cycles are displayed in Figure 8. The discharge capacity of the electrode decrease rapidly during the first 100 cycles, then keep constant after 100 cycles, and lead to 84% retention of the primary capacity after 1000 cycles.

The physical properties, such as thickness, conductivity, and discharge capacity of the HA/graphite hybrid material electrodes with different stoichiometry are found in Table 2. The conductivity of the HA/graphite (2/1, 4/1, 7/1, 10/1, w/w) hybrid material electrodes are 159 S·m^−1^, 49 S·m^−1^, 6.3 S·m^−1^, and 0.34 S·m^−1^, respectively, decreasing dramatically along with the increasing mass ratio of HA/graphite (from 1.17 to 4.85). The larger mass ratio of HA/graphite causes smaller conductivity of the hybrid material electrodes, which can be attributed to the insulating properties of HA. However, the discharge capacity of the hybrid material electrodes are not affected strongly by their conductivity, even for electrodes with conductivity of 0.34 S·m^−1^. We note that the obtained capacity of the hybrid material electrodes are only weakly dependent on the HA/graphite stoichiometry.

## 4. Conclusions

Herein, we utilize the redox reactions of HA—a ubiquitous, sustainable and low cost material—to fabricate electrodes, combining HA with graphite. A mechanochemical method, followed by an aqueous processing step, allows the biocarbon (HA) and carbon (graphite) to meet and form the HA/graphite hybrid material electrodes. During the solvent-free ball milling process, HA helps to exfoliate graphite into few-layer graphene sheets with smaller crystallite size. The HA/graphite hybrid electrodes display a conductivity of up to 160 S·m^−1^ and a discharge capacity of 20 mAhg^−1^. The presented methodology is promising for scalable, sustainable, and low cost production of biocarbon derived electrodes. 

## Figures and Tables

**Table 1:** Comparison of the charge storage performance of some carbon electrodes.

Carbon Electrodes	Charge Storage Performance
HA/graphite hybrid material electrodes	60 F·g^−1^ (20 mAhg^−1^)
Graphite electrode [28]	5 F·g^−1^
Carbon black [29]	12.6 F·g^−1^
Single-walled carbon nanotube [30]	18 F·g^−1^
Poly(3,4-ethylenedioxythiophene) (PEDOT)/Lignin hybrid materials [3]	34 mAhg^−1^
Lignin/reduced graphene oxides hybrid materials [31]	72 mAhg^−1^
Kraft lignin/conductive carbon materials [32]	80 mAhg^−1^

**Table 2:** Physical properties of the HA/graphite hybrid material electrodes with different stoichiometry.

	Thickness/(µm)	Conductivity/(S·m^−1^)	Discharge Capacity/(mAhg^−1^)	Mass Ratio of HA/Graphite in Pellets
HA/graphite (2/1) ^1^	4.1 ± 0.1	159 ± 12	16.7 ± 0.5	1.17
HA/graphite (4/1)	4.2 ± 0.1	49 ± 2	20.7 ± 2.0	2.21
HA/graphite (7/1)	3.0 ± 0.1	6.3 ± 0.4	20.1 ± 0.9	3.78
HA/graphite (10/1)	15.9 ± 0.3	0.34 ± 0.05	18.9 ± 1.6	4.85

^1^ stoichiometry of HA and graphite, w/w.

## Supplementary Materials

The following are available online at https://www.mdpi.com/1996-1944/12/24/4032/s1, stoichiometry calculation of the HA/graphite hybrid material electrodes based on TGA results.
