# Supplementary material for: Biocarbon Meets Carbon—Humic Acid/Graphite Electrodes Formed by Mechanochemistry
Source: Materials (Basel). 2019 Dec 4;12(24):4032. doi: 10.3390/ma12244032 (PMC6947187; doi:10.3390/ma12244032)
Supplement: Supplementary file 1 [file materials-12-04032-s001.pdf]

**Supplementary Materials:** Stoichiometry calculation of the HA/graphite hybrid material electrodes based on TGA results.

The HA/graphite hybrid material electrodes are mixtures of HA and graphite. We assume the final residual mass of HA and graphite, individually, in the HA/graphite hybrid material electrodes are consistent with the pure HA and graphite, respectively, after heating. For example, the residual mass fraction of the pure HA powder after heating is 44.5%, accordingly, for the HA within the hybrid material electrodes after heating, the residual mass fraction is also 44.5%. It is the same condition with graphite. Apart from that, the total mass loss of a HA/graphite hybrid electrode after heating is equal to the mass loss of HA plus the mass loss of graphite in the HA/graphite hybrid electrode, respectively.

Based on the above hypothesis, we set the HA/graphite (2/1, w/w) hybrid material electrode as an example for the stoichiometry calculation. The HA/graphite (2/1, w/w) hybrid material electrode, HA powder and the pristine graphite display a residual mass fraction of 68.2%, 44.5%, and 96.0%, respectively. The amount of HA and graphite in the HA/graphite (2/1, w/w) hybrid electrode before heating are designated as "$x$" and "$y$", respectively, and below we get the equation S1 and S2:

$$x \cdot (1 - 0.445) + y \cdot (1 - 0.960) = (x + y)\ (1 - 0.682), \qquad \text{(S1)}$$

The ratio between x and y is:

$$x/y = (0.96 - 0.682) \ / \ (0.682 - 0.445) = 1.17, \qquad \text{(S2)}$$

Similarly, the stoichiometry of the other HA/graphite (4/1, 7/1, 10/1, w/w) hybrid material electrodes are calculated as 2.21, 3.78, and 4.85, respectively.
